# Genetic Diversity and Excretion Kinetics of Enteroviruses Excreted by Patients with Primary Immunodeficiency in Tunisia over a Five-Year Period (2020–2024)

**DOI:** 10.3390/microorganisms14020329

**Published:** 2026-01-30

**Authors:** Imene Ben Salem, Haifa Khemiri, Marwa Khedhiri, Najla Mekki, Marie-Line Joffret, Nadia Driss, Ilhem Ben Fraj, Monia Ben Khaled, Ines Ben Mrad, Mohamed-Ridha Barbouche, Henda Touzi, Zina Meddeb, Monia Ouederni, Maël Bessaud, Imen Ben Mustapha, Henda Triki, Sondes Haddad-Boubaker

**Affiliations:** 1 Laboratory of Clinical Virology, WHO Regional Reference Laboratory for Poliomyelitis and Measles for the EMR, Institute Pasteur of Tunis, 13 Place Pasteur, le Belvedere, Tunis 1002, Tunisia; haifa.khemiri@pasteur.utm.tn (H.K.); marwa.kehidiri@gmail.com (M.K.); touzihenda@yahoo.fr (H.T.); zinamedeb@gmail.com (Z.M.); henda.triki@pasteur.tn (H.T.); 2 Laboratory of Virus, Host and Vectors (LR 20 IPT 02), Institute Pasteur of Tunis, University of Tunis El Manar, Tunis 1002, Tunisia; 3 Laboratory of Transmission, Control and Immunology of Infections (LR 16 IPT 02), Department of Immunobiology of Infections, Institut Pasteur de Tunis, University of Tunis El Manar, Tunis 1002, Tunisia; najla.mekki@fmt.utm.tn (N.M.); imen.benmustapha@fmt.utm.tn (I.B.M.); 4 Faculty of Medicine, University of Tunis El Manar, Tunis 1002, Tunisia; benfraj-ilhem@live.fr (I.B.F.); moniabkhaled@yahoo.fr (M.B.K.); monia.ouederni@fmt.utm.tn (M.O.); 5 Virus Sensing and Signaling Unit, CNRS UMR 3569, Institut Pasteur, Université de Paris Cité, 75006 Paris, France; mljoffret@gmail.com (M.-L.J.); mael.bessaud@pasteur.fr (M.B.); 6 Laboratoire Associé Au Centre National de Référence Entérovirus/Paréchovirus, 75015 Paris, France; 7 National Program of Immunization, Primary Healthcare Directorate, Ministry of Health, Tunis 1002, Tunisia; nadiadrisschaari@gmail.com (N.D.); ines.benmrad@rns.tn (I.B.M.); 8 National Bone Marrow Transplant Center, Tunis 1002, Tunisia; 9 Department of Microbiology, Immunology and Infectious Diseases, College of Medicine and Medical Sciences, Arabian Gulf University, Manama 26671, Bahrain; ridha.barbouche@pasteur.rns.tn

**Keywords:** Enterovirus, next-generation sequencing, phylogeny, recombination, excetion kinetics

## Abstract

Enteroviruses (EVs) are small, non-enveloped RNA viruses that can cause diverse clinical outcomes, particularly severe in patients with primary immunodeficiency (PID) due to their impaired ability to clear infections. This study aimed to characterize EV excretion among 138 Tunisian PID patients over a five-year period, to identify circulating EV serotypes and assess their genetic diversity. A total of 558 stool samples were collected and analyzed by virus isolation and intratypic differentiation using RT-qPCR. Molecular typing was performed through Sanger sequencing of the VP1 region and whole genome sequencing using Next-Generation Sequencing (NGS) technologies. Phylogenetic analysis was conducted using the Maximum Likelihood (ML) method. EVs were detected in 55 stool samples from 23 patients. The excretion kinetics of EVs ranged between 30 and 946 days. Thirteen serotypes were identified, including one Poliovirus (PV) and twelve Non-Polio Enteroviruses (NPEVs), predominantly belonging to species B. Two previously unreported serotypes in Tunisia were detected: Coxsackievirus A5 (CVA5) and Echovirus type 19 (E19). In addition, five patients presented enhanced susceptibility to the excretion of successive EV serotypes, and one patient exhibited a co-infection. A possible recombination event was identified in one patient involving Coxsackievirus B5 (CVB5), Coxsackievirus A9 (CVA9) and Coxsackievirus B1 (CVB1) sequences. Phylogenetic analysis showed close genetic relationships with European, American and Asian strains. These findings underscore the dynamic nature of EV circulation and the importance of ongoing molecular surveillance to detect emerging serotypes and guide public health strategies.

## 1. Introduction

In the framework of the Global Polio Eradication Initiative (GPEI), surveillance activity of Poliovirus (PV) is based on the investigation of Polio suspected cases presenting with Acute Flaccid Paralysis (AFP), environmental surveillance in wastewater and surveillance of PV and Non-Polio Enterovirus (NPEV) excretion in patients with primary immunodeficiencies (PID) [1,2].

The genus *Enterovirus* (EV) is classified within the *Picornaviridae* family [3], which comprises small, non-enveloped viruses [4]. They are characterized by icosahedral symmetry and a single-stranded positive-sense RNA genome of approximately 7500 nucleotides in length [5]. The genus EV consists of 15 species that share common virion structure, genomic organization and replication mechanisms. Typically, enteroviral infections result in mild symptoms, but more severe clinical manifestations can arise, including myocarditis, AFP and various clinical disorders, particularly with PID patients who are at increased risk of developing recurrent, severe and life-threatening infections [6].

PIDs represent a genetically wide spectrum of disorders that affect specific components of the innate and adaptive immune system [7,8]. The overview of PID cases in Tunisia reveals an important prevalence influenced by the high consanguinity rate observed both in the country and across the Middle East and North Africa (MENA) region [9,10].

EV excretion among PID patients poses a significant challenge, as prolonged viral shedding in these individuals was documented. For instance, a study in India revealed that while the general prevalence of EV shedding in pediatric patients with PID was relatively low, one patient diagnosed with leaky Severe Combined Immunodeficiency (SCID) exhibited a long-term shedding of type 3 Immunodeficiency-Associated Vaccine-Derived PV (iVDPV) over a two-year period [11]. Notably, a patient from the UK with Common Variable Immunodeficiency (CVID) excreted type 2 VDPV for over 28 years [12]. These long-term excretors can harbor divergent PV strains and serve as reservoirs for neurovirulent PV strains that could compromise the efforts of the GPEI [13].

In Tunisia, despite decades of being a Polio-free country, PID individuals remain at a serious risk of persistent EV excretion, especially linked to the use of an oral PV vaccine (OPV). Research published in 2024 revealed the excretion of iVDPVs type 1 and 3 for periods of 134 and 271 days in 2009 and 2019, respectively, in patients with MHC class II deficiency. Rigorous genomic characterization of excreted strains revealed important intra-host diversity and recombinant strains excreted by patients with fatal outcomes [14]. In addition, a previous study carried out in 2012 showed that PID patients present an increased susceptibility to infections with NPEVs. Most cases resolved the excretion within weeks. The longest excretion period was detected in an MHC class II-deficient patient who excreted Echovirus type 11 (E11) over 528 days [15].

The epidemiology of NPEVs in Tunisia is characterized by fluctuation throughout the year, with periods of low and high transmission. Studies conducted between 1992 and 2017 reported frequent isolation of Echoviruses types 6, 11 and 30 [15,16,17]. Other serotypes showed cyclic occurrence, such as Coxsackieviruses type B (CVB), and others have been isolated episodically, mainly Coxsackieviruses type A (CVA) [16]. For PVs, the last cases of paralysis due to wild PV occurred in 1992, and the last wild isolate was detected in 1994. Since then, all PV isolates detected have been Sabin-like (SL) [18] or vaccine-derived [14,15].

In Tunisia, surveillance of EV excretion in PID patients was introduced in November 2023 as part of the National PV surveillance program, complementing the existing AFP surveillance. This study was conducted within the framework of the PID surveillance implementation. The studies carried out between 2005 and 2012 [15,16] provided partial insights into EV circulation among this population, and the present one expands the scope by providing a thorough follow-up of all Tunisian patients with PID, collected from the 24 Tunisian governorates, aiming to identify different EV serotypes, study their excretion kinetics and assess the genetic diversity of circulating strains.

## 2. Materials and Methods

### 2.1. Ethical Statement

This study was ethically approved by the Biomedical Ethics Committee of Pasteur Institute of Tunis (2019/20/I/LR161IPT/V1). Written informed consent was obtained from all patients or their legal guardians. Patient confidentiality was strictly maintained throughout the research process in accordance with the Tunisian and International ethical guidelines and legal requirements.

### 2.2. Studied Population and Sampling

A total of 138 Tunisian patients diagnosed with various types of PID received clinical follow-up over a 5-year period, from September 2020 to December 2024. These patients originated from all Tunisian governorates and presented Predominantly Antibody Deficiencies (PADs) such as Agammaglobulinemia, Common Variable Immunodeficiency (CVID) or Hyper-IgM syndrome (HIGM), and Combined Immunodeficiencies (CIDs) including Severe Combined Immunodeficiency (SCID) and MHC class II deficiency.

A total of 558 fecal specimens were collected in accordance with a meticulously established algorithm in collaboration with the World Health Organization (WHO). The study protocol includes a six-month follow-up for patients who do not excrete EVs, and a monthly follow-up for excretors, continuing until two consecutive negative results are obtained. The samples are transported to the Laboratory of Clinical Virology at Pasteur Institute of Tunis within a timeframe of 24 to 48 h at a temperature of +4 °C. Upon arrival, they are processed within 24 to 48 h and, thereafter, conserved at −20 °C for long-term storage. The integrity of the results is ensured through rigorous adherence to WHO standard protocols [19].

### 2.3. Virus Isolation in Cell Culture

Virus detection was carried out through specimen inoculation on cell culture, following the WHO standard protocols [19]. Stool suspensions were prepared and inoculated onto two distinct cell lines: RD cells (Human Rhabdomyosarcoma) and L20B cells (transgenic mouse cell line expressing the gene encoding the human cellular receptor for PV). The inoculated cells were incubated at 37 °C with daily monitoring for 10 days. The presence of a PV is suspected when a cytopathic effect (CPE) appears on L20B and RD cell lines, while NPEVs generally induce a positive culture result only in RD cell lines.

### 2.4. Intratypic Differentiation of Polioviruses

PVs, showing a CPE on L20B and RD cell lines, were further identified using intratypic differentiation (ITD), a multiplex real-time PCR enabling the identification of the serotypes of PV strains as well as their vaccine or wild origin, using the rRT-PCR 5.2 ITD kit (CDC, Atlanta, GA, USA) according to the manufacturer’s instructions.

### 2.5. Typing of NPEVs

NPEVs were typed using whole genome sequencing (WGS) and partial sequencing of the VP1 region when a WGS could not be obtained. Viral RNA extraction was performed using the QIAamp viral RNA mini kit (Qiagen, Germany) in adherence to the manufacturer’s instructions. All RNA extracts were either immediately used or stored at −20 °C for subsequent use.

Out of the 558 stool samples analyzed, 55 tested positive for EV and underwent WGS using Next-Generation Sequencing (NGS) technologies, notably Oxford Nanopore MinIon (Oxford Nanopore Technologies, Oxford, UK) and Illumina (Illumina inc., San Diego, CA, USA), or partially sequenced targeting the VP1 region with Sanger technology (Applied Biosystems, Foster City, CA, USA).

#### 2.5.1. WGS Using Oxford Nanopore MinIon Technology

The extracted RNAs were reverse transcribed to cDNA using the LunaScript^TM^ RT SuperMix kit (New England Biolabs, Ipswich, MA, USA). The obtained cDNA was then amplified using 2 pools of “in-house” primers (Supplementary Table S1) targeting multiple regions of the EV genome. The resulting gene pools were purified using AMPure XP magnetic beads (Beckman Coulter, Brea, CA, USA) prior to quantification with the Qubit dsDNA High Sensitivity (HS) Assay Kit (Thermo Fisher Scientific, Waltham, MA, USA) to ensure optimal quality for DNA end repair and barcoding. Barcodes were ligated to the samples using Native Barcoding Expansion (Oxford Nanopore Technologies, Oxford, UK ). Following this step, the barcoded samples were pooled and purified using AMPure XP magnetic beads (Beckman Coulter, CA, USA). Adapters ligation was subsequently carried out, using Adapter Mix II (AMII) (New England Biolabs, MA, USA), followed by an additional purification step to ensure the removal of any unbound adapters and quantification. Finally, the prepared DNA was injected into the flow cell after checking on the number of pores available, to generate EV genome sequences on the MinIon MK1C sequencer (Oxford Nanopore Technologies, Oxford, UK ). Sequencing data were analyzed using Geneious Prime v2025.0.2 following a defined workflow. The analysis included read trimming to remove low-quality bases and adapters, mapping of filtered reads to EV reference genomes for consensus sequence reconstruction and genome coverage assessment, and annotation to identify functional regions.

#### 2.5.2. WGS Using Illumina Technology

Illumina technology (Illumina inc., San Diego, CA, USA) was used to perform EV-positive genomes sequencing, following a previously described protocol. Briefly, for each specimen, the two PCR products were pooled and subsequently purified employing a vacuum-based approach, after which they were transferred to the sequencing platform PIBNet (Pasteur International Bioresources Network, Institut Pasteur Paris). The libraries were generated utilizing 1 ng of DNA with the Nextera XT DNA Library Preparation kit in a SureCycler 8800 thermocycler (Agilent Technologies, Santa Clara, CA, USA). Subsequent to purification on AMPure beads (Beckman Coulter, CA, USA), library quality was assessed using the High Sensitivity D1000 assay (Agilent Technologies) on a TapeStation 2200. Sequencing was carried out on an Illumina NextSeq HiSeq adopting a read depth of 50× (Illumina Inc., San Diego, CA, USA). All procedures followed the manufacturer’s instructions. Data analysis was carried out using CLC Genomics Workbench 8.5 to pair reads and assemble contigs from the raw sequencing data. Subsequently, de novo assembly was performed using CLC Main Workbench [20].

#### 2.5.3. Partial Sequencing in the VP1 Region and Sequence Analysis

RNA extracts were processed by RT-PCR according to a previously described “in-house” protocol, using primers designed by Bailly et al. targeting the VP1-coding region of the genome [21], which is the major antigenic protein and the gold standard for EV typing [22].

The PCR products are purified using the QIAquick PCR kit (Qiagen, Dusseldorf, Germany) and subsequently sequenced bidirectionally on an ABI 3130 automated DNA sequencer (Applied Biosystem, ABI 3130, CA, USA) using the ABI PRISM BigDye Terminator Cycle Sequencing Ready Process kit (Invitrogen, CA, USA). The sequence data were subjected to analysis with Molecular Evolutionary Genetic Analysis (MEGA) software v7.0.26. The serotype was determined by comparing the acquired sequences to sequences available in the GenBank database. The Tunisian sequences were submitted to the GenBank database, and accession numbers are provided in the Supplementary Table S2.

### 2.6. Recombination Analysis

Full genome sequences were analyzed for potential recombination breakpoints using SimPlot Software v3.5.1, with default parameters (step size: 20 bp; GapStrip: on; 100 replicates; Kimura 2-parameter model; T/t ratio: 2.0; Neighbor-joining method).

### 2.7. Phylogeny

Due to the limited availability of complete EV genome sequences in the GenBank database and to ensure broad global representation of EV circulation, only the complete VP1 nucleotide sequences were used for the phylogenetic analysis. Sequences of interest were retrieved from the GenBank database (Date of access: January 2025) and covered a temporal range from 1991 to 2024. The duplicated ones were eliminated using the BLASTclust command “-i infile.fasta -o outfile.txt -p F -L 1 -S 99 -b T” to generate a non-redundant set of sequences. Thereafter, all sequences were aligned using the Mafft program “Multiple Alignment using Fast Fourier Transform” (https://mafft.cbrc.jp/alignment/server/index.html). Phylogenetic trees were constructed using MEGA Software v7.0.26. An appropriate outgroup sequence (Accession number: AY184219.1) was included to root the tree. The evolutionary history was inferred using the Maximum Likelihood (ML) approach, with a repetitive bootstrap number of 1000. Following tree reconstruction, sequences that were identical and originated from the same geographic location or were collected in the same or closely related years were further excluded to minimize sampling bias and temporal overrepresentation. The final number of VP1 sequences retained for each serotype is detailed in Table 1, ensuring a balanced and representative dataset for phylogenetic inference.

## 3. Results

### 3.1. Demographic and Clinical Data of EV-Positive Patients

The studied cohort comprised children (<15 years) (*n* = 106) as well as teenagers and adults (*n* = 32) with a median age of 11 years and a male predominance: 75 males (54%) vs. 63 females (46%). The investigated patients were diagnosed with various forms of PID: 95 patients had Predominantly Antibody Deficiencies (PAD), and 43 had Combined Immunodeficiencies (CIDs). PAD included Agammaglobulinemia (*n* = 33), Common Variable Immunodeficiency (CVID) (*n* = 43) and Hyper-IgM syndrome (HIGM) (*n* = 19). CID were classified into Severe Combined Immunodeficiency (SCID) (*n* = 13), MHC class II deficiency (*n* = 18) and other types of CID (*n* = 12). The distribution of PID patients is illustrated in Table 2.

Among the 138 PID patients, 23 tested positive for EV infection (17%), including one PV excreter and 22 patients shedding NPEVs. The EV-positive group showed a male-to-female ratio of 1.5 (14/9) and a median age of 6 years. Notably, five adult patients were identified as NPEV excreters, with ages spanning from 21 to 58 years. These EV-positive patients provided a total of 55 fecal specimens, corresponding to an overall positivity rate of 9.85%.

**Figure 1:**
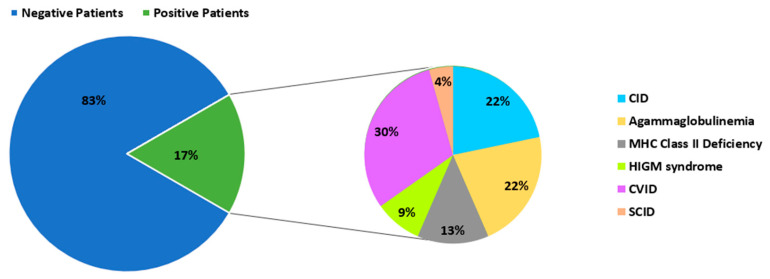
Repartition of PV- and NPEV-positive patients among different PID groups.

**Figure 2:**
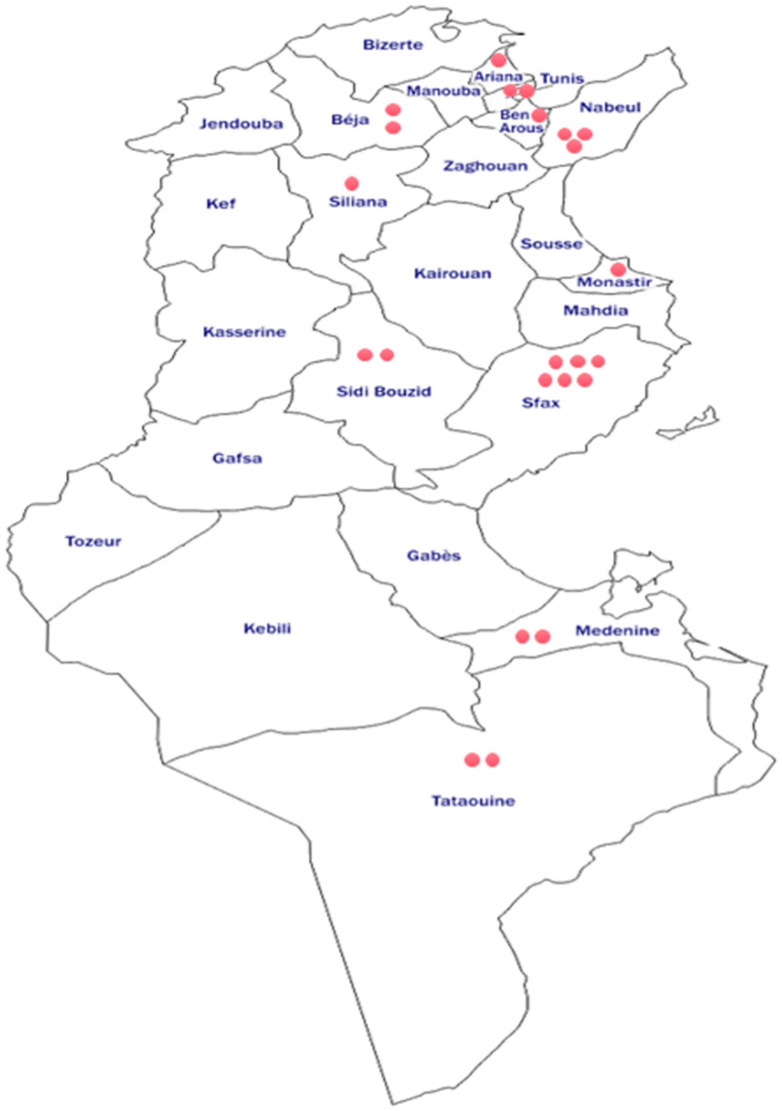
Demographic distribution of EV excretors. Note: each point corresponds to one patient.

Within the various categories of PIDs, patients with CVID, including the single PV-positive case, accounted for 30% of EV-positive patients (*n* = 23). Agammaglobulinemia and CID each represented 22% of EV-positive cases, followed by MHC class II deficiency (13%). In contrast, HIGM (9%) and SCID (4%) were the least represented groups (Figure 1). Regarding EV infection rates within each PID category, CID patients exhibited the highest positivity rate (41.7%) (5/12), followed by patients with MHC class II deficiency (16.7%) (3/18), CVID (16.3%) (7/43), and Agammaglobulinemia (15.2%) (5/33). Lower EV positivity rates were observed among patients with HIGM (10.5%) (2/19) and SCID (1/13) (7.7.%). However, Fisher’s exact test showed no statistically significant association between PID subtype and EV positivity (*p* > 0.05). Geographically, the majority of excreters originated from coastal governorates, with the highest rates reported in Sfax (26%) and Nabeul (13%) (Figure 2).

### 3.2. Serotype Diversity

**Figure 3:**
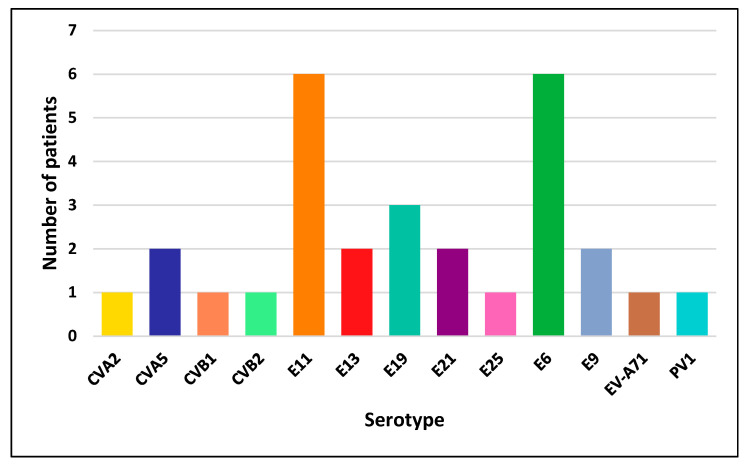
Distribution of Enterovirus serotypes excreted by the EV-positive patients, CVA2 = Coxsackievirus A2, CVA5 = Coxsackievirus A5, CVB1 = Coxsackievirus B1, CVB2 = Coxsackievirus B2, E11 = Echovirus 11, E13 = Echovirus 13, E19 = Echovirus 19, E21 = Echovirus 21, E25 = Echovirus 25, E6 = Echovirus 6, E9 = Echovirus 9, EV-A71 = Enterovirus A71, and PV1 = Poliovirus 1.

A total of 13 distinct serotypes were isolated among the EV-positive patients. The group EV-B was the most prevalent, including Echoviruses type 13, 11, 19, 21, 25, 6, 9 and Coxsackievirus B2. The group EV-A, which includes Coxsackieviruses A2 and A5 and EV-A71, was less common, similarly to EV-C, represented only by PV1. Overall, Echoviruses type 11 and 6 were the most frequently observed, involving six patients for each serotype, followed by Echovirus type 19 (three patients) and Echoviruses type 13, 21 and E9 and Coxsackievirus A5 (two patients) for each serotype. Other serotypes, including Coxsackievirus A2, Coxsackieviruses B1-2, Echovirus type 25, EV-A71 and PV1, were detected less commonly in only one patient (Figure 3). Importantly, some patients excreted more than one serotype during the study period; therefore, they were counted in more than one category. Consequently, the numbers shown reflect the total number of patients excreting each serotype, and not the number of unique patients.

### 3.3. Excretion Kinetics of EVs

Among the 23 EV excreters identified herein, the majority (*n* = 20) exhibited short-term viral excretion (defined as an excretion period shorter than 6 months), with shedding periods ranging from 30 days (one month) to 114 days (approximately 4 months). Among them, one patient excreted PV for a period of one month.

Two patients showed prolonged excretion of EVs for periods exceeding 6 months. Patient 3 excreted Echovirus type 11 for 242 days. Patient 2 represented an exceptional case with prolonged excretion of Echovirus type 13 for up to 946 days (more than 31 months), representing the longest shedding period recorded in this cohort.

**Figure 4:**
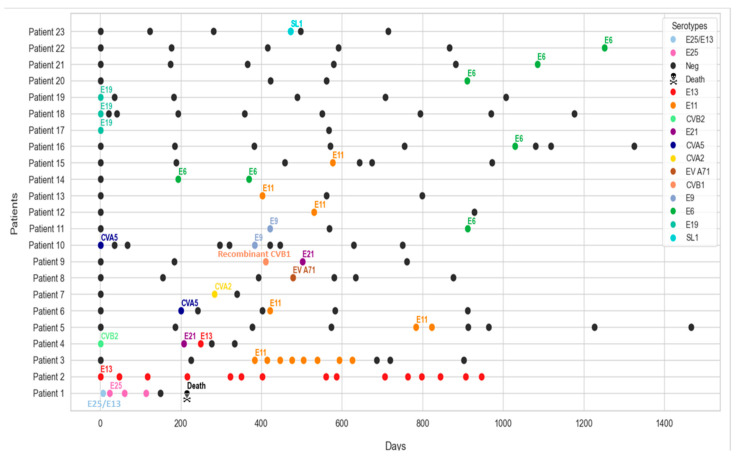
Excretion kinetics of EVs.

Five patients presented enhanced susceptibility to the excretion of multiple EV serotypes. Patient 4, with CID, excreted several serotypes, of which Echoviruses types 13 and 21, and Coxsackievirus B2 (CVB2) over a period of 249 days (approximately 8 months). Additionally, patients 6, 9, 10 and 11 were infected with multiple EV serotypes during the study period at different time points. Notably, co-infections were reported. Indeed, patient 1 experienced a co-infection with two different serotypes, Echoviruses type 25 and 13, on day 7. Thereafter, he continued to excrete only Echovirus type 25 until virus clearance and death after a shedding period of 114 days (Figure 4).

### 3.4. Genetic Characterization of PV Strain

The comparison of the obtained Tunisian strain with the reference strain under accession number AY184219.1 revealed the accumulation of eight mutations, as illustrated in Supplementary Figure S1. This corresponds to a mutation rate of 0.9%, which is below the threshold defining VDPV, consequently confirming that the Tunisian isolate is classified as Sabin-like and not a Vaccine-Derived Poliovirus (VDPV). This divergence rate corresponds to a possible excretion period of approximately 11 months or a potentially important mutation rate attributable to the immunodeficiency context. Remarkably, the identified mutations were not located within any of the known antigenic sites.

### 3.5. Recombination Event

**Figure 5:**
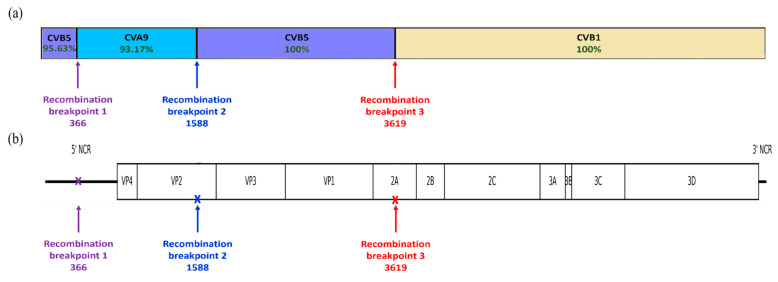
Schematic representation of the recombination event: (**a**) recombination breakpoints across the EV genome revealing CVB5-CVA9-CVB1 mosaic pattern and (**b**) genomic positions of recombination breakpoints.

Only one sample obtained on day 411 from patient 9 exhibited evidence of possible recombination events. Simplot analysis suggested the possible occurrence of recombination involving CVB5, CVB1 and CVA9 sequences (Figure 5a). The best match obtained with Basic Local Alignment Search Tool (BLAST) analysis indicated that the 5′ region (nt 1–366) showed 95.63% nucleotide identity with a CVB5 strain (accession number: MG845891.1). The first recombination breakpoint was identified at nt 366, marking the transition from CVB5 to CVA9. The intermediate fragment spanning nt 367–1588 showed 93.17% nucleotide identity with CVA9 (accession number: OR678417.1). A second recombination breakpoint was detected at nt 1588, within the VP2 region, followed by a fragment from 1589 to 3619 showing 100% nucleotide identity with the CVB5 strain (accession number: MG845891.1). The third recombination breakpoint was located at nt 3619 within the 2A region (Figure 5b), after which the region extending from nt 3619 onward showed 100% nucleotide identity with a CVB1 strain (accession number: NC_001472.1). This recombination pattern was consistent with the Bootscan analysis, which revealed a clear shift in similarity profiles corresponding to the identified breakpoints (Supplementary Figure S2).

### 3.6. Genetic Diversity of NPEV Isolates

To investigate the genetic diversity of NPEVs, the complete VP1 sequences were considered.

**Figure 6:**
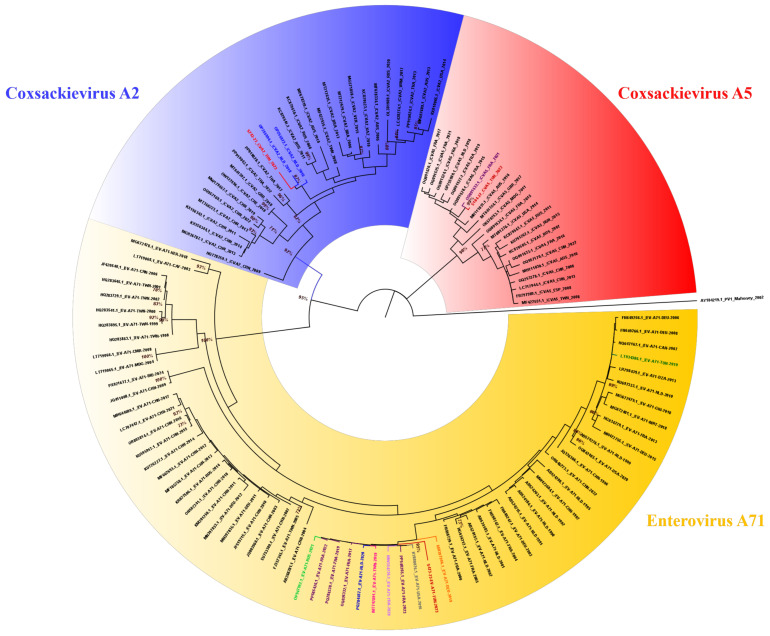
Phylogenetic tree generated with EV-A sequences and a reference sequence (Mahoney, AY184219.1) as an out-group. The red branches correspond to the Tunisian strains. The most genetically associated sequences are represented in blue (the Netherlands, NLD), purple (France, FRA), orange (Germany, DEU), gray (the United States of America, USA), light green (Russia, RUS), lilac (Thailand, THA) and pink (Taiwan, TWN). The green branches correspond to the Tunisian sequences previously reported in the GenBank database. Coxsackievirus A5 is highlighted in red, Coxsackievirus A2 is highlighted in blue and Enterovirus A71 is highlighted in yellow.

Phylogenetic analysis of EV-A sequences (Figure 6, Supplementary Figure S4) was performed using 22 CVA5, 27 CVA2 and 66 EV-A71 sequences retrieved from the GenBank database (Supplementary Table S3). All Tunisian sequences from CVA5 and CVA2 clustered into groups of European sequences from different countries, supported by a robust bootstrap value of 99% and 87%, respectively. On the other hand, the Tunisian EV-A71 sequence isolated in 2023 belongs to genogroup E (Supplementary Figure S3) and is grouped with European, Asian and American (USA) strains isolated between 2016 and 2023. This cluster is strongly supported by a robust bootstrap value of 95%. In contrast, it was genetically distant from a previously reported Tunisian strain isolated in 2019. This phylogenetic pattern underscores the genetic heterogeneity of EV-A71 circulating strains and suggests different independent introduction events to Tunisia rather than autochthonous virus circulation.

**Figure 7:**
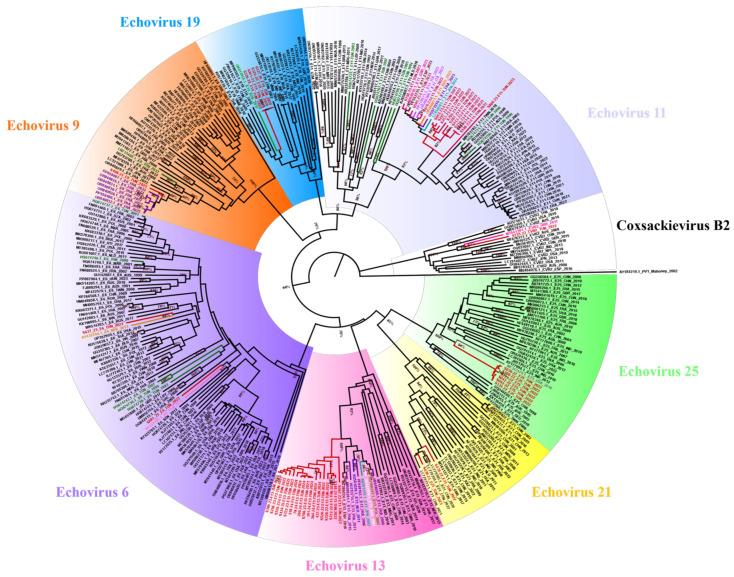
Phylogenetic tree generated with EV-B sequences and a reference sequence (Mahoney, AY184219.1) as an out-group. The red branches correspond to the Tunisian strains. The most genetically associated sequences are represented in blue (the Netherlands, NLD), purple (France, FRA), orange (Germany, DEU), gray (the United States of America, USA), light green (Russia, RUS), lilac (Italy, ITA), light blue (Japan, JPN), dark red (Spain, ESP), pink (the United Kingdom, GBR), brown (China, CHN), olive green (Poland, POL), white (Australia, AUS), light pink (Senegal, SEN) and camel (Niger, NER). The green branches correspond to the Tunisian sequences previously reported in the GenBank database. Echovirus 11 is highlighted in gray, Coxsackievirus B2 is highlighted in white, Echovirus 25 is highlighted in green, Echovirus 21 is highlighted in yellow, Echovirus 13 is highlighted in pink, Echovirus 6 is highlighted in purple, Echovirus 9 is highlighted in orange and Echovirus 19 is highlighted in blue.

Phylogenetic analysis of EV-B sequences (Figure 7, Supplementary Figure S5) was performed using a dataset comprising 75 E11 sequences, 16 CVB2 sequences, 39 E25 sequences, 26 E21 sequences, 37 E13 sequences, 89 E6 sequences, 40 E9 sequences and 22 E19 sequences (Supplementary Table S4). Tunisian E25 sequences grouped closely with a U.S strain detected in 2016, supported with a robust bootstrap value of 94%. Tunisian CVB2, E9 and E19 sequences clustered with European sequences. E6 clustered with European and African sequences. Similarly, Tunisian E21 and E11 sequences clustered with both European and Asian sequences, with a robust bootstrap value of 96% and 83%, respectively. Notably, the Tunisian strains showed genetic divergence from previously reported Tunisian sequences, suggesting the introduction of new viral lineages or evolutionary divergence over time.

## 4. Discussion

This study provides a comprehensive overview of PV and NPEV excretion among all patients with PID recorded across the 24 Tunisian governorates over a five-year period from 2020 to 2024. Our objectives are to shed light on viral excretion dynamics, assess serotype diversity and describe epidemiological patterns of excreted PVs and NPEVs.

A total of 138 Tunisian PID patients were enrolled in this study, including 95 patients with PAD (Predominantly Antibody Deficiency) and 43 with CID (Combined Immunodeficiency), as both conditions result in immunoglobulin deficiencies and can be associated with prolonged viral excretion. In PAD, the absence or dysfunction of B cells leads to impaired production of neutralizing antibodies, which are crucial for viral clearance. In contrast, CID affects both T- and B-cell compartments, compromising not only antibody production but also cytotoxic T cell-mediated viral elimination.

Among patients, 23 (17%) tested positive for EV infections, with a median age of 6 years, including five adults (22%) aged between 21 and 58 years. In a previous study conducted in 2012 and targeting 82 PID patients, 11 EV excreters were reported, including 2 adults aged 24 and 26 [15]. Indeed, the persistent immune system dysfunction can predispose adults to chronic or recurrent EV infections. Thus, it is relevant, in the framework of PID surveillance, to investigate all age groups in contrast to AFP, which may concern only children younger than 15 years. Interestingly, the geographic distribution of EV-positive patients revealed a clustering in the governorates of Sfax (26%) and Nabeul (13%). This finding aligns with previous national surveillance data reporting higher EV circulation in urban and coastal regions, likely reflecting increased population density and improved access to healthcare facilities. It may also be linked to higher rates of PID in these areas in association with the important reported consanguinity rates [16].

Among the EV-positive patients, CVID accounted for the largest proportion of cases (30%), followed by Agammaglobulinemia and CID (22% each). However, the proportion of EV-positive cases alone does not reflect true differences in susceptibility to infection. When infection rates were analyzed within each PID category, patients with CID exhibited the highest EV-positivity rate (41.7%), suggesting a potentially increased susceptibility to EV infections in this group. Nevertheless, given the limited number of patients in PID categories, these findings should be interpreted with caution, as the statistical power to detect significant intergroup differences remains limited. These observations are at odds with earlier reports, such as the study elaborated by Mohanty et al. in India, where CVID stands out as the most common type of PID among EV-positive patients (6 patients out of 29) [23]. This disparity can be the result of variations in cohort composition, diagnostic procedures, or surveillance strategies across populations. The relatively low rates of EVs infection observed in patients with SCID (4%) and HIGM (9%) may be linked to reduced life expectancy, as these patients rarely survive beyond one year of age. Earlier diagnosis and hematopoietic stem cell transplantation can improve the prognosis and survival of these patients [15,24].

The detection of only a single case of SL1 PV, for a possible period of 11 months, aligns with the Tunisian patterns. The circulation of VDPVs remains limited, and only SL strains have become predominant as a result of sustained high levels of vaccination coverage [25]. The latest cases of iVDPVs excreters identified in Tunisia, through surveillance of AFP, were received in 2009, 2016 and 2019 from three PID patients with MHC class II deficiency, with an excretion duration ranging from 44 to 271 days [14]. The infection rate of NPEV (17%) in our study was higher than in previous national studies. In 2012, the EV infection rate among PID patients was 13.4% [15]. In addition, in an 11-year period study conducted between 2007 and 2017, the prevalence of PV and NPEV in PID patients was 6.8% and 12.4%, respectively [17]. This increase could be linked to the enhanced surveillance programs, the quality of the samples, in addition to the intensified screening of high-risk groups.

The distribution of the 12 NPEV serotypes identified in our study highlights the important EVs diversity in our country, with the detection of two previously unreported serotypes: Coxsackievirus A5 (CVA5) (*n* = 2) and Echovirus type 19 (E19) (*n* = 3) [16,17,26]. This may reflect the natural diversity of EV circulation patterns as well as the importance of ongoing molecular surveillance in the detection of uncommon serotypes that may go unnoticed. The predominance of species B among the identified NPEVs in the present study was reported in other countries in Africa, Asia and Europe [27,28,29]. Echoviruses type 11, 6 and 19 were more frequently isolated. In contrast, other serotypes were sporadically isolated during the study course, including Coxsackieviruses A2 and B1-2, as well as Echoviruses type 25 and EV-A71. These trends reflect the inherent instability of EV epidemiology, driven by factors such as herd immunity changes and global population movements. Large-scale outbreaks in Europe and Asia have demonstrated that changes in population-level immunity can result in the replacement of serotypes or the reappearance of previously rare EV types [30,31]. In addition, a possible recombination event involving CVB5, CVB1, and CVA9 was identified with an important nucleotide identity percentage (≥95%). This genomic configuration suggests sequential recombination events between EV-B and EV-A serotypes, reinforcing the well-established role of recombination as a major evolutionary mechanism within the EV-A and B species. These findings are consistent with previous studies reporting inter- and intra-serotypic recombination among circulating CVB and CVA9 strains, which may contribute to genetic diversification, viral fitness, and the emergence of novel variants [32]. These patterns underscore the critical need for strengthened EV surveillance and tailored management approaches in PID populations, in light of evolving immunization policies and regional disparities in healthcare infrastructure. This shift in the epidemiological landscape, especially after the eradication of types 2 and 3 wild PVs (WPV) [33], creates a favorable environment for the transmission and circulation of NPEVs [31,34].

The investigation of EV excretion kinetics in patients with PID revealed short-term excretion among the majority of the excreters (20 out of 23), with viral clearance occurring within a limited time frame. Nevertheless, two patients exhibited prolonged excretion lasting about 8 months. Patient 3, diagnosed with Agammaglobulinemia, excreted Echovirus type 11 for 242. Atypically, patient 2, diagnosed with CVID, excreted Echovirus type 13 for an extended period up to 946 days (approximately 31 months). In fact, there has been an association between CVID and long-term shedding of EVs. Notably, all chronic EV excretors recorded in the WHO registry have been diagnosed with CVID [35]. This finding is anticipated, given that CVID consists of a broad spectrum of associated pathologies and longer life expectancy compared to other types of PIDs, which contribute to the likelihood of prolonged EV excretion [36,37]. In Tunisia, the longest recorded shedding period of EVs was reported in a 4-year-old child with MHC class II deficiency, who excreted Echovirus type 21 for 588 days [15]. There have been reports of prolonged EV shedding in pediatric patients with PID globally, with the duration varying depending on individual immune status. One of the extremely chronic shedding periods involved a child diagnosed with CVID in the United Kingdom, who excreted iVDPV2 for over 28 years, the longest duration ever reported [12]. In Iran, prolonged iVDPV2 shedding has also been observed for a period lasting up to 720 days [13]. Additionally, an Indian patient with SCID was identified to have shed type 3 iVDPV for two years [11].

Several patients in our cohort (*n* = 6) exhibited shedding of numerous EV serotypes either consecutively or simultaneously. This high EV susceptibility may be attributed to impaired immune responses to clear infections or continuous exposure within a high-transmission environment. Notably, patient 1 showed concomitant infection with Echoviruses type 25 and 13 during the first week of infection, before virus clearance 114 days later, and death. Similarly, patients 4, 6, 9, 10, and 11 experienced multiple shedding episodes. Particularly, patients 6 and 11 excreted various EV serotypes over time, suggesting possible reinfection, viral reactivation, or prolonged subclinical persistence. This pattern of recurrent or overlapping EV infections has also been reported in previous studies involving PID patients [38,39]. In addition, patients were more prone to be vulnerable to the excretion of EV-B group serotypes, including predominantly Echoviruses type 6, 11 and 19. Several factors may account for the higher frequency of these serotypes. In fact, the EV-B group is known to circulate widely in many countries and is frequently associated with both gastrointestinal and systemic infections. As a result, patients are likely to experience repeated exposure. Furthermore, EV-B viruses have been reported to display efficient replication in intestinal epithelial cells and may possess specific determinants that facilitate persistence and prolonged shedding. Immunodeficiency also plays a crucial role. In patients with PID, viral clearance may be delayed, allowing these serotypes to be detected more often than others. Overall, the important detection of E6, E11 and E19 probably reflects an interplay of virological, immunological and epidemiological factors rather than a single mechanism [40,41]

In order to further characterize the isolated NPEV serotypes and determine their indigenous or imported origin, a phylogenetic analysis was carried out for all serotypes. The phylogenetic pattern indicates multiple and diverse introductions of NPEV strains from different geographic origins over time, especially of European, African, American and Asian origin [42,43]. This could be explained by the important commercial, cultural and tourist exchanges between Tunisia, the European countries and certain Asian nations. The EV-A71 strain identified in Tunisia was found to belong to genogroup E. This genogroup has previously been reported only in West and Central Africa, including Niger, Cameroon, the Central African Republic, Senegal, Guinea, and Mauritania, suggesting that genogroup E may be indigenous to the African continent. The detection of this lineage in Tunisia expands its known geographic range and highlights the potential for wider circulation of this genogroup across Africa [44]. Moreover, the absence of a distinct clade including Tunisian sequences suggests the lack of continuous endemic circulation within the country and reflects sporadic importation events rather than persistent local transmission. This highlights the dynamic nature of EV circulation, likely influenced by human mobility and regional connectivity.

## 5. Conclusions

In conclusion, the present study provides valuable insights into the excretion kinetics and the molecular diversity of EVs in Tunisia, thereby advancing our understanding of their genetic epidemiology and phylogenetic relationships. Through the molecular characterization and comparative analysis of circulating strains, this study provides an additional perspective on the dynamics of EV circulation across the country. These results enhance the current national and international database of EVs and provide a valuable foundation for upcoming epidemiological surveillance, molecular investigations, and public health strategies aimed at monitoring and controlling EV infections in Tunisia.

## Figures and Tables

**Table 1:** Number of sequences retained for phylogenetic analysis by serotype.

Genus	Serotype	Number of Sequences
Enterovirus A	Coxsackievirus A5	22
	Coxsackievirus A2	27
	Enterovirus A71	66
Enterovirus B	Echovirus 11	75
	Coxsackievirus B2	16
	Echovirus 25	39
	Echovirus 21	26
	Echovirus 13	37
	Echovirus 6	89
	Echovirus 9	40
	Echovirus 19	22

**Table 2:** Distribution of different categories of PID among the 138 patients included in the study.

Type of PID	Negative Patients	PV-Positive Patients	NPEV-Positive Patients	Total
Predominantly Antibody Deficiency (PAD)				
Agammaglobulinemia	28	0	5	33
Common Variable Immunodeficiency (CVID)	36	1	6	43
Hyper-IgM Syndrome (HIGM)	17	0	2	19
Combined Immunodeficiency (CID)				
Severe Combined Immunodeficiency (SCID)	12	0	1	13
MHC Class II Deficiency	15	0	3	18
CID	7	0	5	12
Total	115	1	22	138

PV = Poliovirus; NPEV= Non-Polio Enterovirus.

## Data Availability

The original contributions presented in this study are included in the article/Supplementary Materials. Further inquiries can be directed to the corresponding author.

## Supplementary Materials

The following supporting information can be downloaded at: https://www.mdpi.com/article/10.3390/microorganisms14020329/s1, Figure S1: Mutation positions obtained by alignment of the reference sequence and the isolate consensus sequence; Figure S2: Recombination event between CVB5, CVA9 and CVB1 sequences using SimPlot software; Figure S3: Phylogenetic tree of EV-A71 sequences; Figure S4: Phylogenetic tree generated with EV-A sequences and a reference sequence (Mahoney, AY184219.1) as an out-group; Figure S5: Phylogenetic tree generated with EV-B sequences and a reference sequence (Mahoney, AY184219.1) as an out-group; Table S1: Characteristics of in-house designed primer pools of EV-A, EV-B and EV-C for whole-genome sequencing; Table S2: Accession numbers of Tunisian sequences; Table S3: List of nucleotide sequences of EV-A retrieved from GenBank, including their accession numbers, collection dates and geographic origins; Table S4: List of nucleotide sequences of EV-B retrieved from GenBank, including their accession numbers, collection dates and geographic origins.

## Abbreviations

The following abbreviations are used in this manuscript:

WPV	Wild Poliovirus
GPEI	Global Polio Eradication Initiative
PV	Poliovirus
AFP	Acute Flaccid Paralysis
NPEV	Non-Polio Enterovirus
EV	Enterovirus
PID	Primary Immunodeficiency
OPV	Oral Polio Vaccine
MENA	Middle East and North Africa
SCID	Severe Combined Immunodeficiency
CVID	Common Variable Immunodeficiency
iVDPV	Immunodeficiency-Associated Vaccine-Derived Poliovirus
VDPV	Vaccine-Derived Poliovirus
E	Echovirus
CVA	Coxsackievirus A
CVB	Coxsackievirus B
SL	Sabin-Like
PAD	Predominantly Antibody Deficiency
HIGM	Hyper-IgM Syndrome
CID	Combined Immunodeficiency
WHO	World Health Organization
ITD	Intratypic Differentiation
CPE	Cytopathic Effect
WGS	Whole-genome Sequencing
NGS	New-Generation Sequencing
PIBNet	Pasteur International Bioresources Network
MEGA	Molecular Evolutionary Genetic Analysis
Mafft	Multiple Alignment using Fast Fourier Transformer
ML	Maximum Likelihood
BLAST	Basic Local Alignment Search Tool

## References

[1] Global Polio Eradication Initiative. (n.d.). Global Polio Eradication—Eradicate Polio Now. https://www.polioeradication.org/.

[2] Chumakov K., Kew O. (2014). The Poliovirus Eradication Initiative.

[3] Oberste M.S., Pallansch M.A. (2016). Enteroviruses and Parechoviruses. Clinical Virology Manual.

[4] Wang S., Wang K., Zhao K., Hua S., Du J. (2020). The structure, function, and mechanisms of action of enterovirus non-structural protein 2C. Front. Microbiol..

[5] Cifuente J.O., Moratorio G. (2019). Evolutionary and Structural overview of human picornavirus CaPSID antibody evasion. Front. Cell. Infect. Microbiol..

[6] Modrow S., Falke D., Truyen U., Schätzl H. (2013). Viruses with Single-Stranded, Positive-Sense RNA Genomes.

[7] Li L., Ivanova O., Driss N., Tiongco-Recto M., Da Silva R., Shahmahmoodi S., Sazzad H.M.S., Mach O., Kahn A., Sutter R.W. (2014). Poliovirus excretion among persons with primary immune deficiency disorders: Summary of a Seven-Country Study series. J. Infect. Dis..

[8] Geha R.S., Notarangelo L.D., Casanova J., Chapel H., Conley M.E., Fischer A., Hammarström L., Nonoyama S., Ochs H.D., Puck J.M. (2007). Primary immunodeficiency diseases: An update from the International Union of Immunological Societies Primary Immunodeficiency Diseases Classification Committee. J. Allergy Clin. Immunol..

[9] Barbouche M., Galal N., Ben-Mustapha I., Jeddane L., Mellouli F., Ailal F., Bejaoui M., Boutros J., Marsafy A., Bousfiha A.A. (2011). Primary immunodeficiencies in highly consanguineous North African populations. Ann. N. Y. Acad. Sci..

[10] Mellouli F., Mustapha I.B., Khaled M.B., Besbes H., Ouederni M., Mekki N., Ali M.B., Larguèche B., Hachicha M., Sfar T. (2015). Report of the Tunisian Registry of Primary Immunodeficiencies: 25-Years of Experience (1988–2012). J. Clin. Immunol..

[11] Mohanty M.C., Madkaikar M.R., Desai M., Taur P., Nalavade U.P., Sharma D.K., Gupta M., Dalvi A., Shabrish S., Kulkarni M. (2017). Poliovirus Excretion in Children with Primary Immunodeficiency Disorders, India. Emerg. Infect. Dis..

[12] Dunn G., Klapsa D., Wilton T., Stone L., Minor P.D., Martin J. (2015). Twenty-Eight years of Poliovirus Replication in an Immunodeficient Individual: Impact on the Global Polio Eradication Initiative. PLoS Pathog..

[13] Aghamohammadi A., Abolhassani H., Kutukculer N., Wassilak S.G., Pallansch M.A., Kluglein S., Quinn J., Sutter R.W., Wang X., Sanal O. (2017). Patients with Primary Immunodeficiencies Are a Reservoir of Poliovirus and a Risk to Polio Eradication. Front. Immunol..

[14] Salem I.B., Khemiri H., Drechsel O., Arbi M., Böttcher S., Mekki N., Fraj I.B., Souiai O., Yahyaoui M., Farhat E.B. (2024). Reversion of neurovirulent mutations, recombination and high intra-host diversity in vaccine-derived poliovirus excreted by patients with primary immune deficiency. J. Med. Virol..

[15] Driss N., Ben-Mustapha I., Mellouli F., Yahia A.B., Touzi H., Bejaoui M., Ghorbel M.B., Triki H., Barbouche M. (2012). High Susceptibility for Enterovirus Infection and Virus Excretion Features in Tunisian Patients with Primary Immunodeficiencies. Clin. Vaccine Immunol..

[16] Bahri O., Rezig D., Nejma-Oueslati B.B., Yahia A.B., Sassi J.B., Hogga N., Sadraoui A., Triki H. (2005). Enteroviruses in Tunisia: Virological surveillance over 12 years (1992–2003). J. Med. Microbiol..

[17] Chouikha A., Rezig D., Driss N., Abdelkhalek I., Yahia A.B., Touzi H., Meddeb Z., Farhat E.B., Yahyaoui M., Triki H. (2021). Circulation and Molecular Epidemiology of Enteroviruses in Paralyzed, Immunodeficient and Healthy Individuals in Tunisia, a Country with a Polio-Free Status for Decades. Viruses.

[18] Haddad-Boubaker S., Ben Yahia A., Bahri O., Morel V., Balanant J., Delpeyroux F., Triki H. (2008). Genetic features of polioviruses isolated in Tunisia, 1991–2006. J. Clin. Virol..

[19] WHO (2004). Polio Laboratory Manual.

[20] Joffret M., Polston P.M., Razafindratsimandresy R., Bessaud M., Heraud J., Delpeyroux F. (2018). Whole genome sequencing of enteroviruses species A to D by High-Throughput Sequencing: Application for viral mixtures. Front. Microbiol..

[21] Bailly J., Brosson D., Archimbaud C., Chambon M., Henquell C., Peigue-Lafeuille H. (2002). Genetic diversity of echovirus 30 during a meningitis outbreak, demonstrated by direct molecular typing from cerebrospinal fluid. J. Med. Virol..

[22] Harvala H., Broberg E., Benschop K., Berginc N., Ladhani S., Susi P., Christiansen C., McKenna J., Allen D., Makiello P. (2018). Recommendations for enterovirus diagnostics and characterisation within and beyond Europe. J. Clin. Virol..

[23] Mohanty M.C., Desai M., Mohammad A., Aggarwal A., Govindaraj G., Bhattad S., Lashkari H.P., Rajasekhar L., Verma H., Kumar A. (2023). Assessment of Enterovirus Excretion and Identification of VDPVs in Patients with Primary Immunodeficiency in India: Outcome of ICMR–WHO Collaborative Study Phase-I. Vaccines.

[24] Estivariz C.F., Krow-Lucal E.R., Mach O. (2024). Immunodeficiency-Related Vaccine-Derived Poliovirus (IVDPV) Infections: A Review of Epidemiology and Progress in Detection and Management. Pathogens.

[25] Haddad-Boubaker S., Ould Mohamed-Abdallah M.V., Ben Yahia A., Triki H. (2010). Genetic recombination in vaccine poliovirus: Comparative study in strains excreted in course of vaccination by oral poliovirus vaccine and circulating strains. Pathol. Biol..

[26] Rmadi Y., Elargoubi A., González-Sanz R., Mastouri M., Cabrerizo M., Aouni M. (2022). Molecular characterization of enterovirus detected in cerebrospinal fluid and wastewater samples in Monastir, Tunisia, 2014–2017. Virol. J..

[27] Fernandez-Garcia M.D., Kebe O., Fall A.D., Ndiaye K. (2017). Identification and molecular characterization of non-polio enteroviruses from children with acute flaccid paralysis in West Africa, 2013–2014. Sci. Rep..

[28] Yang X., Wu Y., Zhao H., Liu P., Liang L., Yin A. (2024). Emergence and circulation of enterovirus B species in infants in southern China: A multicenter retrospective analysis. Virulence.

[29] De Schrijver S., Vanhulle E., Ingenbleek A., Alexakis L., Johannesen C.K., Broberg E.K., Harvala H., Fischer T.K., Benschop K.S.M., Albert J. (2025). Epidemiological and clinical insights into enterovirus circulation in Europe, 2018–2023: A multi-center retrospective surveillance study. J. Infect. Dis..

[30] Harvala H., Griffiths M., Solomon T., Simmonds P. (2014). Distinct systemic and central nervous system disease patterns in enterovirus and parechovirus infected children. J. Infect..

[31] Pons-Salort M., Parker E.P., Grassly N.C. (2015). The epidemiology of non-polio enteroviruses. Curr. Opin. Infect. Dis..

[32] Combelas N., Holmblat B., Joffret M.L., Colbère-Garapin F., Delpeyroux F. (2011). Recombination between poliovirus and coxsackie A viruses of species C: A model of viral genetic plasticity and emergence. Viruses.

[33] Hsu C.H., Kader M., Mahamud A., Bullard K., Jorba J., Agbor J., Safi M.M., Jafari H.S., Ehrhardt D. (2019). Progress toward Poliomyelitis Eradication—Pakistan, January 2018–September 2019. MMWR Morb. Mortal. Wkly. Rep..

[34] Brown D.M., Zhang Y., Scheuermann R.H. (2020). Epidemiology and Sequence-Based Evolutionary analysis of circulating Non-Polio enteroviruses. Microorganisms.

[35] Macklin G., Liao Y., Takane M., Dooling K., Gilmour S., Mach O., Kew O.M., Sutter R.W. (2017). Prolonged Excretion of Poliovirus among Individuals with Primary Immunodeficiency Disorder: An Analysis of the World Health Organization Registry. Front. Immunol..

[36] Quinti I., Soresina A., Spadaro G., Martino S., Donnanno S., Agostini C., Claudio P., Franco D., Pesce A.M., Borghese F. (2007). Long-Term Follow-Up and Outcome of a Large Cohort of Patients with Common Variable Immunodeficiency. J. Clin. Immunol..

[37] Tebbens R.J.D., Pallansch M.A., Thompson K.M. (2015). Modeling the prevalence of immunodeficiency-associated long-term vaccine-derived poliovirus excretors and the potential benefits of antiviral drugs. BMC Infect. Dis..

[38] Ruffner M.A., Sullivan K.E., Henrickson S.E. (2017). Recurrent and sustained viral infections in primary immunodeficiencies. Front. Immunol..

[39] Makimaa H., Ingle H., Baldridge M.T. (2020). Enteric viral co-infections: Pathogenesis and perspective. Viruses.

[40] Laassri M., Zagorodnyaya T., Hassin-Baer S., Handsher R., Sofer D., Weil M., Karagiannis K., Simonyan V., Chumakov K., Shulman L. (2018). Evolution of echovirus 11 in a chronically infected immunodeficient patient. PLoS Pathog..

[41] Genoni A., Canducci F., Rossi A., Broccolo F., Chumakov K., Bono G., Salerno-Uriarte J., Salvatoni A., Pugliese A., Toniolo A. (2017). Revealing enterovirus infection in chronic human disorders: An integrated diagnostic approach. Sci. Rep..

[42] Fares W., Rezig D., Seghier M., Yahia A.B., Touzi H., Triki H. (2011). Phylogenetic analysis of complete VP1 sequences of echoviruses 11 and 6: High genetic diversity and circulation of genotypes with a wide geographical and temporal range. J. Med. Microbiol..

[43] Othman I., Mirand A., Slama I., Mastouri M., Peigue-Lafeuille H., Aouni M., Bailly J. (2015). Enterovirus Migration Patterns between France and Tunisia. PLoS ONE.

[44] Fernandez-Garcia M., Volle R., Joffret M., Sadeuh-Mba S., Gouandjika-Vasilache I., Kebe O., Wiley M.R., Majumdar M., Simon-Loriere E., Sakuntabhai A. (2018). Genetic Characterization of Enterovirus A71 Circulating in Africa. Emerg. Infect. Dis..

