# Supplementary material for: Genetic Diversity and Excretion Kinetics of Enteroviruses Excreted by Patients with Primary Immunodeficiency in Tunisia over a Five-Year Period (2020–2024)
Source: Microorganisms. 2026 Jan 30;14(2):329. doi: 10.3390/microorganisms14020329 (PMC12943416; doi:10.3390/microorganisms14020329)
Supplement: Supplementary file 1 [file microorganisms-14-00329-s001.zip › Supplementary Figure S1.pdf]

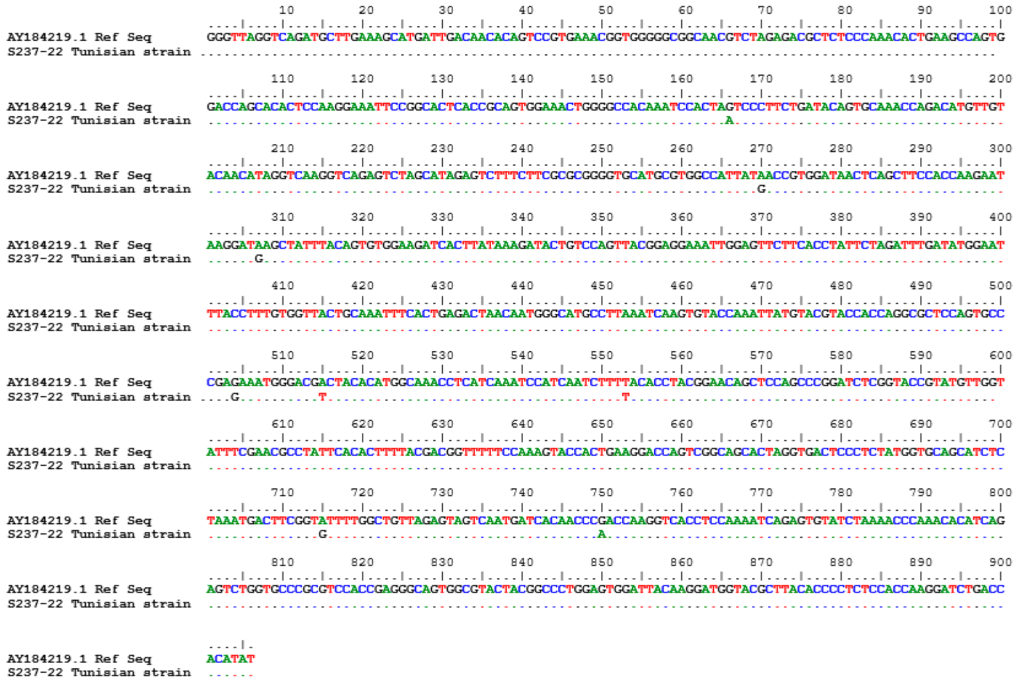

Supplementary Figure S1. Mutation positions obtained by alignment of the reference sequence and the isolate consensus sequence
