# Supplementary material for: Genetic Diversity and Excretion Kinetics of Enteroviruses Excreted by Patients with Primary Immunodeficiency in Tunisia over a Five-Year Period (2020–2024)
Source: Microorganisms. 2026 Jan 30;14(2):329. doi: 10.3390/microorganisms14020329 (PMC12943416; doi:10.3390/microorganisms14020329)
Supplement: Supplementary file 1 [file microorganisms-14-00329-s001.zip › Supplementary Figure S2.pdf]

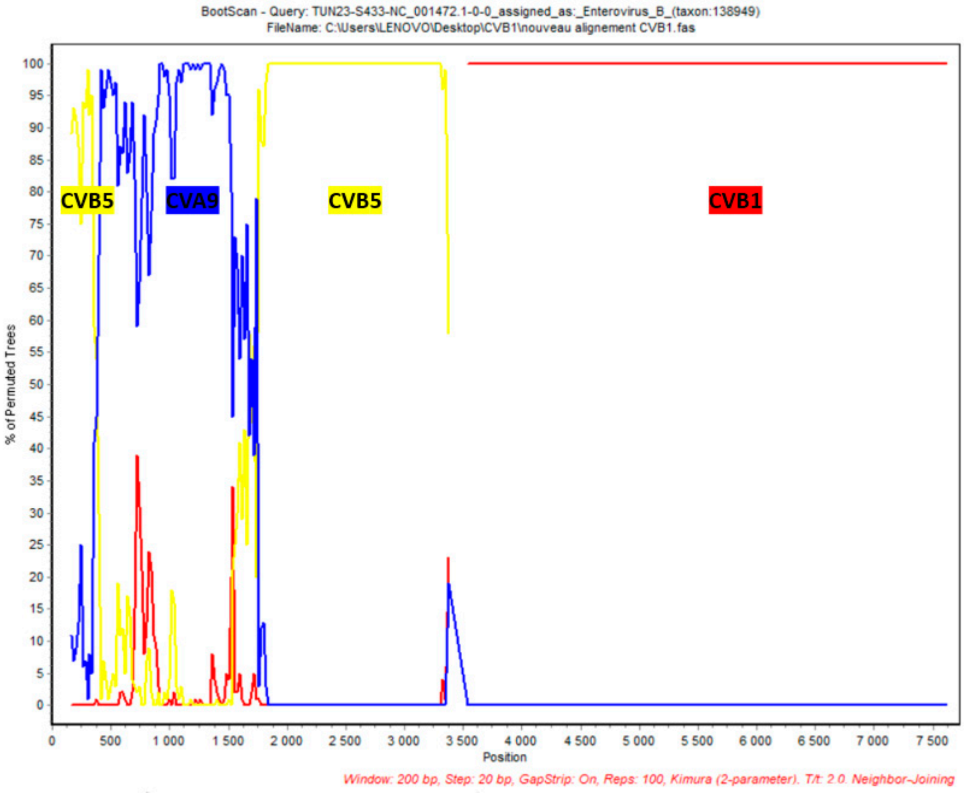

Supplementary Figure S2. Recombination event between CVB5, CVA9 and CVB1 sequences using SimPlot software

CVB5= Coxsackievirus B5; CVA9= Coxsackievirus A9; CVB1= Coxsackievirus B1
