# Supplementary material for: Genetic Diversity and Excretion Kinetics of Enteroviruses Excreted by Patients with Primary Immunodeficiency in Tunisia over a Five-Year Period (2020–2024)
Source: Microorganisms. 2026 Jan 30;14(2):329. doi: 10.3390/microorganisms14020329 (PMC12943416; doi:10.3390/microorganisms14020329)
Supplement: Supplementary file 1 [file microorganisms-14-00329-s001.zip › Supplementary Figure S4.pdf]

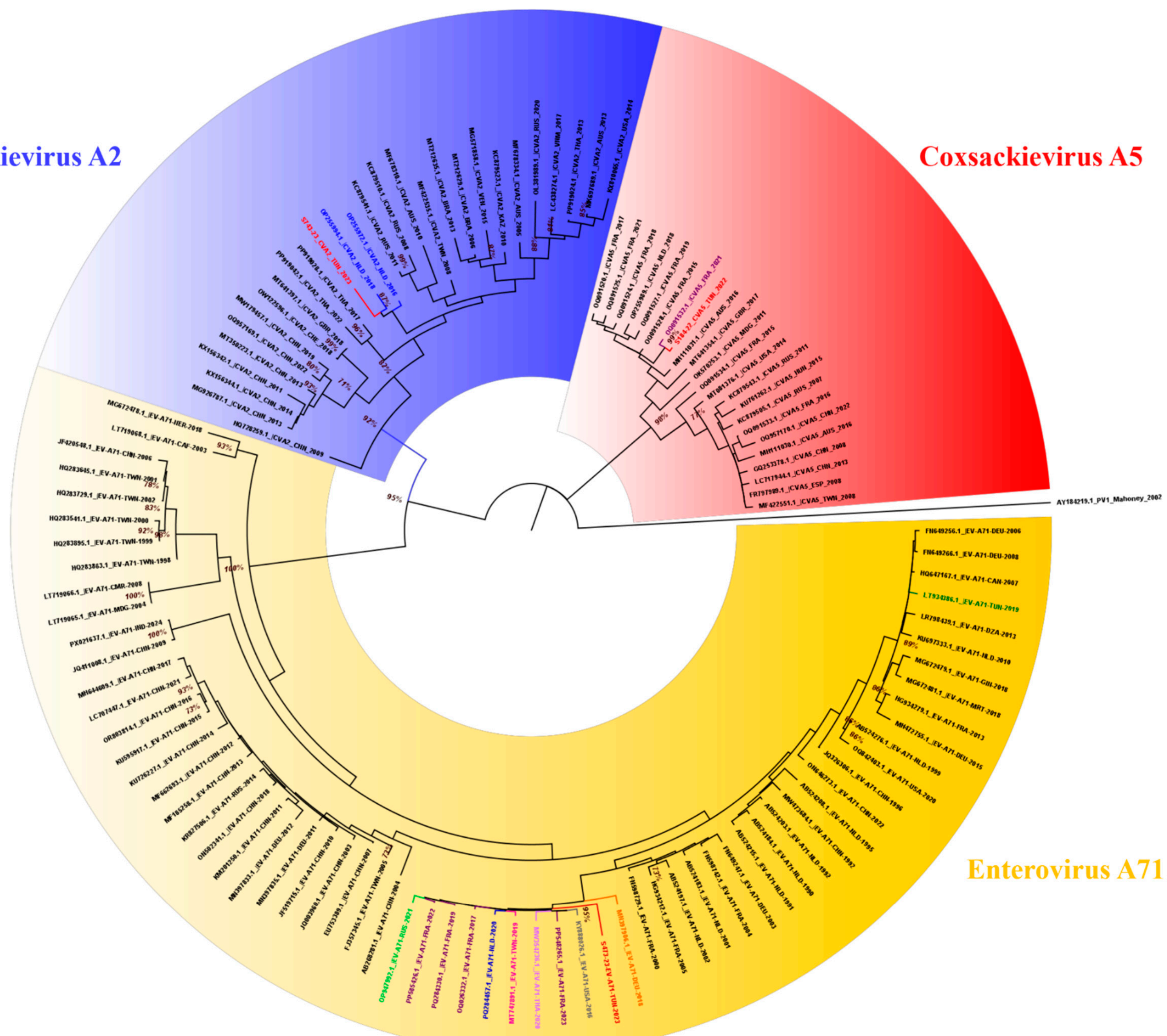

Supplementary Figure S4. Phylogenetic tree generated with EV-A sequences and a reference sequence (Mahoney, AY184219.1) as an out-group. The red branches correspond to the Tunisian strains. The most genetically associated sequences are represented in blue (Netherlands, NLD), purple (France, FRA), orange (Germany, DEU), Grey (United States of America, USA), light green (Russia, RUS), lilac (Thailand, THA) and pink (Taiwan, TWN). The green branches correspond to the Tunisian sequences previously reported in the GenBank database. Coxsackievirus A5 is highlighted in red, Coxsackievirus A2 is highlighted in blue and Enterovirus A71 is highlighted in yellow.
