# Supplementary material for: Genetic Diversity and Excretion Kinetics of Enteroviruses Excreted by Patients with Primary Immunodeficiency in Tunisia over a Five-Year Period (2020–2024)
Source: Microorganisms. 2026 Jan 30;14(2):329. doi: 10.3390/microorganisms14020329 (PMC12943416; doi:10.3390/microorganisms14020329)
Supplement: Supplementary file 1 [file microorganisms-14-00329-s001.zip › Supplementary Figure S5.pdf]

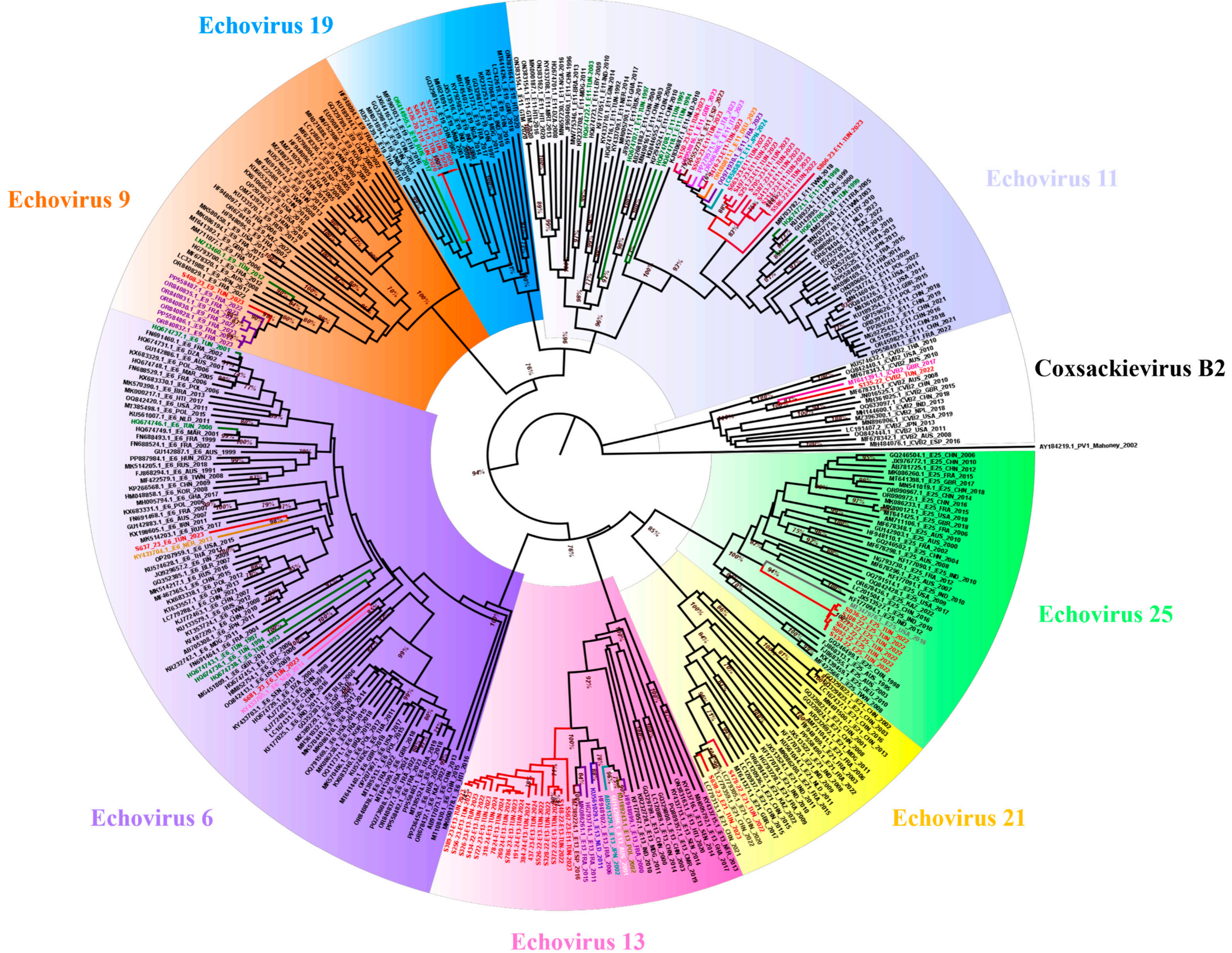
Echovirus 19
Echovirus 11
Echovirus 9
Coxsackievirus B2
Echovirus 25
Echovirus 6
Echovirus 21
Echovirus 13

Supplementary Figure S5. Phylogenetic tree generated with EV-B sequences and a reference sequence (Mahoney, AY184219.1) as an out-group. The red branches correspond to the Tunisian strains. The most genetically associated sequences are represented in blue (Netherlands, NLD), purple (France, FRA), orange (Germany, DEU), Grey (United States of America, USA), light green (Russia, RUS), lilac (Italy, ITA), light blue (Japan, JPN), dark red (Spain, ESP), pink (The United Kingdom, GBR), brown (China, CHN), olive green (Poland, POL), white (Australia, AUS), light pink (Senegal, SEN) and camel (Niger, NER). The green branches correspond to the Tunisian sequences previously reported in the GenBank database. Echovirus 11 is highlighted in grey, Coxsackievirus B2 is highlighted in white, Echovirus 25 is highlighted in green, Echovirus 21 is highlighted in yellow, Echovirus 13 is highlighted in pink, Echovirus 6 is highlighted in purple, Echovirus 9 is highlighted in orange and Echovirus 19 is highlighted in blue.
